# Two triphosphate tunnel metalloenzymes from apple exhibit adenylyl cyclase activity

**DOI:** 10.3389/fpls.2022.992488

**Published:** 2022-10-06

**Authors:** Ye Yuan, Zhiguo Liu, Lili Wang, Lixin Wang, Shuangjiang Chen, Yahong Niu, Xin Zhao, Ping Liu, Mengjun Liu

**Affiliations:** ^1^ College of Horticulture, Hebei Agricultural University, Baoding, China; ^2^ Research Center of Chinese Jujube, Hebei Agricultural University, Baoding, China

**Keywords:** adenylyl cyclase, verification, tertiary structure, triphosphate tunnel metalloenzymes, woody plant

## Abstract

Adenylyl cyclase (AC) is the key catalytic enzyme for the synthesis of 3′,5′-cyclic adenosine monophosphate. Various ACs have been identified in microorganisms and mammals, but studies on plant ACs are still limited. No AC in woody plants has been reported until now. Based on the information on HpAC1, three enzymes were screened out from the woody fruit tree apple, and two of them (MdTTM1 and MdTTM2) were verified and confirmed to display AC activity. Interestingly, in the apple genome, these two genes were annotated as triphosphate tunnel metalloenzymes (TTMs) which were widely found in three superkingdoms of life with multiple substrate specificities and enzymatic activities, especially triphosphate hydrolase. In addition, the predicted structures of these two proteins were parallel, especially of the catalytic tunnel, including conserved domains, motifs, and folded structures. Their tertiary structures exhibited classic TTM properties, like the characteristic EXEXK motif and β-stranded anti-parallel tunnel capable of coordinating divalent cations. Moreover, MdTTM2 and HpAC1 displayed powerful hydrolase activity to triphosphate and restricted AC activity. All of these findings showed that MdTTMs had hydrolysis and AC activity, which could provide new solid evidence for AC distribution in woody plants as well as insights into the relationship between ACs and TTMs.

## Introduction

Adenylyl cyclase (AC) is the only key enzyme responsible for the synthesis of 3′,5′-cyclic adenosine monophosphate (cAMP), while cAMP is an important signaling molecule and the second messenger in animals and lower eukaryotes (Goodman et al., 1970; Gerisch et al., 1975). It has been confirmed to exist in plant tissues (Witters et al., 1996) and contributes to multiple biological processes, such as participating in pollen tube growth and reorientation (Moutinho et al., 2001), influencing flowering (Kessler and Steinberg, 1973), responding to biotic and abiotic stresses (Gao et al., 2012; Ordoñez et al., 2014; Sabetta et al., 2019), promoting tobacco BY-2 cell division (Ehsan et al., 1998), and regulating ion transport with cyclic nucleotide-gated channels (Lemtiri-Chlieh and Berkowitz, 2004; Maathuis and Sanders, 2010; Zelman et al., 2012; Lu et al., 2015; Wang et al., 2020).

A total of six classes of non-homologous ACs have been cloned, functionally verified, and structurally analyzed from mammals and microorganisms (Tresguerres et al., 2011), but the existence of plant ACs was controversial for a time like cAMP (Ashton and Polya, 1978) due to the restricted content of cAMP in plants and the non-availability of orthologous genes categorized as ACs, while the AC activity of ZmPSiP from maize was experimentally verified, which opened the door to identify ACs in plants (Moutinho et al., 2001). Afterward, 14 other enzymes have been identified to provide AC function, as demonstrated by five other herbaceous plants, which are *Marchantia polymorpha* (Kasahara et al., 2016), *Nicotiana tabacum* (Ito et al., 2014), *Hippeastrum* × *hybridum* (Świeżawska et al., 2014), *Brachypodium distachyon* (Świeżawska et al., 2020; Duszyn et al., 2022), and *Arabidopsis thaliana* (Al-Younis et al., 2021). However, the identification of these plant ACs was mainly through three methods. Firstly, almost half of the plant ACs were identified by screening for the core AC catalytic center motif that only consists of a few conserved amino acids involved in catalysis so far, and these are AtClAP (Chatukuta et al., 2018), AtKUP5 (Al-Younis et al., 2018), AtKUP7 (Al-Younis et al., 2015), AtLRR (Bianchet et al., 2019; Ruzvidzo et al., 2019), AtPPR (Ruzvidzo et al., 2013), AtNCED3 (Al-Younis et al., 2021), and AtAC (Sehlabane et al., 2022). Secondly, omics analysis was performed for searching ACs—for example, quantitative proteomics techniques that assisted the discovery of ZmRPP13-LK3 in maize (Yang et al., 2020). The third method was homologous cloning based on the genetic information of acquired ACs—for example, *HpAC1* from *H. hybridum* was obtained through homologous cloning upon noted ACs with designed degenerate primers (Świeżawska et al., 2014).

As plant ACs are not conserved, it is not surprising that previously determined plant ACs rarely succeeded in being classified into any of the six existing classes of ACs. However, MpCAPE-AC and HpAC1 are the only two exceptions. MpCAPE-AC, found in the basal land plant *M. polymorpha*, was classified as a class III AC, while HpAC1 was classified as a class IV AC which belongs to the triphosphate tunnel metalloenzymes (TTMs) superfamily. TTMs are capable of hydrolyzing triphosphates such as thiamine triphosphate, adenosine triphosphate (ATP), and inorganic triphosphates (PPPi) as well as cyclizing ATP like YpAC from the bacteria *Yersinia pestis* (Gallagher et al., 2006; Gallagher et al., 2011) and BdTTM3 (Świeżawska et al., 2020) and HpAC1 from the plants *B. distachyon* and *H. hybridum.* As a result, we speculated that the orthologous proteins in this superfamily might also possess AC activity. Furthermore, all of the confirmed plant ACs were from herbaceous species, with no woody plant ACs discovered so far. So, we, on the basis of HpAC1, attempted to find ACs from woody plants. In the current study, the significant woody fruit tree apple was selected for the cAMP content of apple fruit had been detected (Liu and Wang, 1991). The question was whether the candidate genes had any AC activity and, if so, whether this can possibly provide valuable structural and catalytic information on both ACs and TTMs in plants.

## Results

### Genome-wide identification of candidate ACs from apple

Diverse ACs have been found in mammals and microorganisms, which were categorized into six classes, designated I to VI, while nearly all the ACs in the higher plants which have been identified so far were classified into none of these six classes and HpAC1 from *H. hybridum* was assigned into class IV AC for sharing with YpAC, the CYTH-like_Pase domain that was also known as TTM. However, the CYTH domain not only is ubiquitously distributed in bacterial, archaeal, eukaryal, and botanic taxa but also has exhibited AC activity from some species like *Y. pestis*, *A. hydrophila*, *B. distachyon* and *H. hybridum*. Thus, to attempt and find the orthologous genes of *HpAC1* in the apple (*Malus domestica*) genome, the protein information of HpAC1 was pursued.

**Figure 1:**
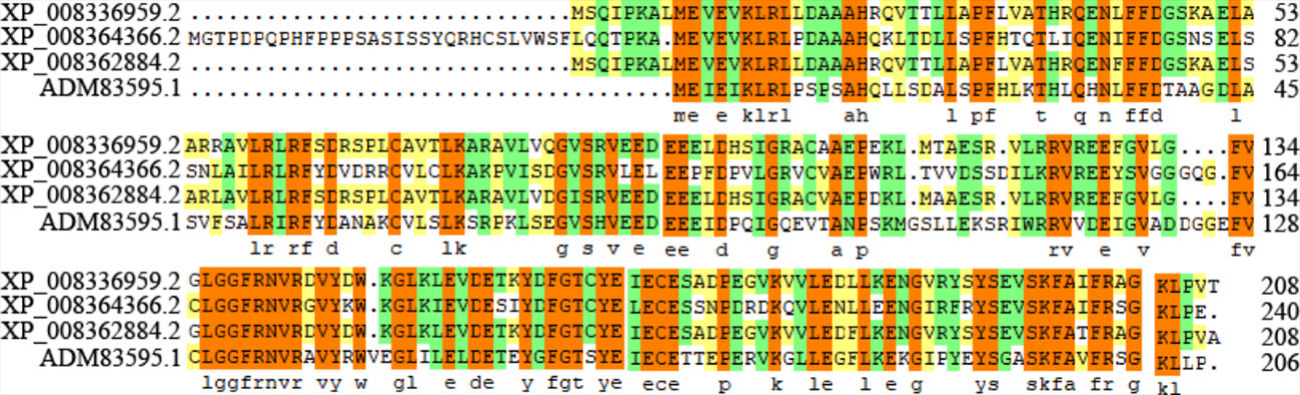
Amino acid sequence alignment analysis of MdTTM1, MdTTM2, MdTTM3, and HpAC1. The alignment analysis was conducted with the software DNAMAN.

**Figure 2:**
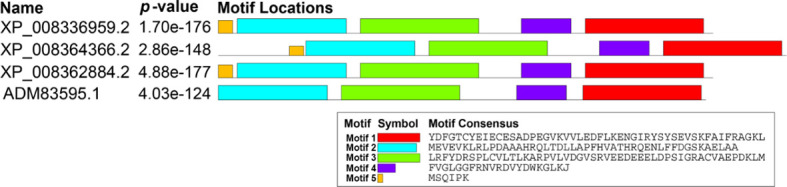
Motifs and conserved domains of MdTTM1, MdTTM2, MdTTM3, and HpAC1. MEME suite was used to identify conserved motifs (https://meme-suite.org/meme/tools/meme).

Three candidates’ ACs were screened out from the apple genome, but interestingly, they were all annotated as TTMs. Then, we designated them as MdTTM1 (LOC103400052), MdTTM2 (LOC103428055), and MdTTM3 (LOC103426573) (**Supplementary Table S1**). The similarity of amino acid sequences among these three candidate ACs from apple and HpAC1 reached 65.65%, including the region not belonging to the CYTH_like domain (**Figure 1**). In addition, their motif structures were similar as well. Through the Multiple Em for Motif Elicitation (MEME) analysis, five conserved motifs of these proteins were obtained (**Figure 2**). Besides this, MdTTMs and HpAC1 shared four of them, and the signature motif EXEXK was found in the second motif of all the MdTTMs with identical amino acids. All these structural analyses of MdTTMs might demonstrate that they probably had AC activities, so both *in vivo* and *in vitro* activities were tested to verify their AC activity consequently.

### AC activity verification of candidate ACs *in vitro*

**Figure 3:**
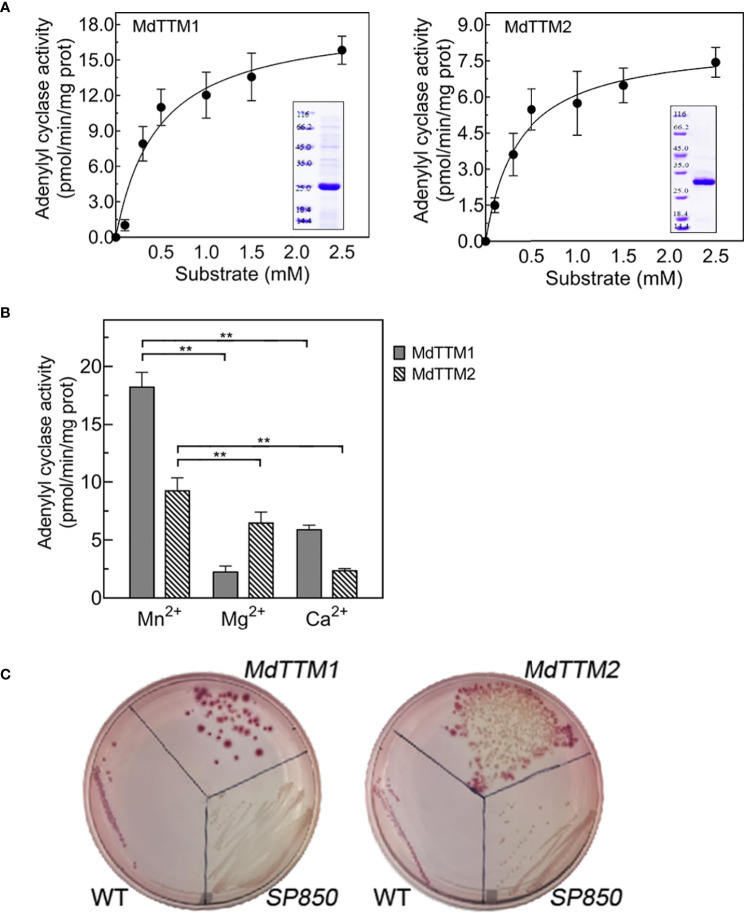
Adenylyl cyclase (AC) activity analysis of MdTTM1 and MdTTM2. **(A)** Enzymatic activity of MdTTM1 and MdTTM2 in response to various concentrations of ATP. The fitted curve was added with GraphPad Prism (ver. 9.4.0). The *R* ^2^ of MdTTM1 and MdTTM2 fitted curves were 0.93 and 0.92, respectively. The icons show the SDS-PAGE analysis of two affinity recombinant proteins. The molecular weight of MdTTM1 was equal to 25 kDa, and that of MdTTM2 was greater than 25 kDa. **(B)** Enzymatic activity of MdTTM1 and MdTTM2 with the support of different divalent cations: Mn^2+^, Mg^2+^, and Ca^2+^. The values are the mean of three independent replicates. Error bars represent standard deviation (**P < 0.01). The catalytic rate was calculated by determining the cAMP generated from a reaction mixture containing 50 μg of purified protein, 2.5 mM divalent cation, and ATP as the substrate. **(C)** Functional complementation of an AC-deficient *E*. *coli cyaA* mutation with MdTTM1 and MdTTM2. Colonies of host cells transformed with a recombinant vector are displayed with red color, while cells harboring an empty vector remain with white color.

To analyze whether the above-mentioned three MdTTMs were capable of displaying AC activity, their enzyme activities were tested *in vitro*. As shown in **Figure 3A**, MdTTM1 and MdTTM2 both displayed AC activity, while MdTTM3 had no AC activity so that we did not do a further analysis on it. In fact, even MdTTM1 and MdTTM2 only represented limited AC activities with *V*_max_ of 18.5 and 8.3 pmol/min/μg, respectively. The optimal reaction conditions that MdTTM1 and MdTTM2 required were similar. They both preferred a neutral pH and a reaction temperature of 30°C (**Supplementary Figure S2**). Besides this, as metalloenzymes, the AC activities of MdTTM1 and MdTTM2 were also related to divalent cations in the reaction system, such as Mn^2+^ than Mg^2+^ and Ca^2+^. As shown in **Figure 3B**, MdTTM1 and MdTTM2 showed a higher AC activity with the support of Mn^2+^ than Mg^2+^ and Ca^2+^; particularly, MdTTM1 displayed a much higher AC activity. It is interesting that, except for Mn^2+^, the AC activity of MdTTM1, MdTTM2, and HpAC1 exhibited divalent cation biases between Mg^2+^ and Ca^2+^. MdTTM1 displayed a lower catalytic efficiency with the assistance of Mg^2+^, while it was a bit higher at Ca^2+^ condition. In comparison, MdTTM2 preferred Mg^2+^ over Ca^2+^. Although Mg^2+^ and Ca^2+^ contributed much less to AC activity than Mn^2+^, the ion biases of MdTTM1 and MdTTM2 seemed to be capable of classifying MdTTM1 and MdTTM2 into two types, according to catalytic intensity, and indicate the structural characteristic differences in ATP catalysis.

### Verification of candidate AC activities *in vivo*

To further verify the AC activities of MdTTM1 and MdTTM2, a complementation experiment was carried out *in vivo*. The SP850 strain of *Escherichia coli* is known as a deletion mutant of endogenous AC (*cyaA*), causing the disability of cAMP synthesis and, afterwards, lactose operon regulation. As shown in **Figure 3C**, heterologously transformed MdTTM1 and MdTTM2 into SP850 strains could form red colonies as well as wild-type *E. coli*, while the SP850 strains with vector could not exploit lactose on glucose shortage culture medium and showed white colonies. These results suggested that these two—MdTTM1 and MdTTM2—could successfully rescue a mutant strain and indeed have AC activity.

### Structural analysis of MdTTM1 and MdTTM2 proteins

**Figure 4:**
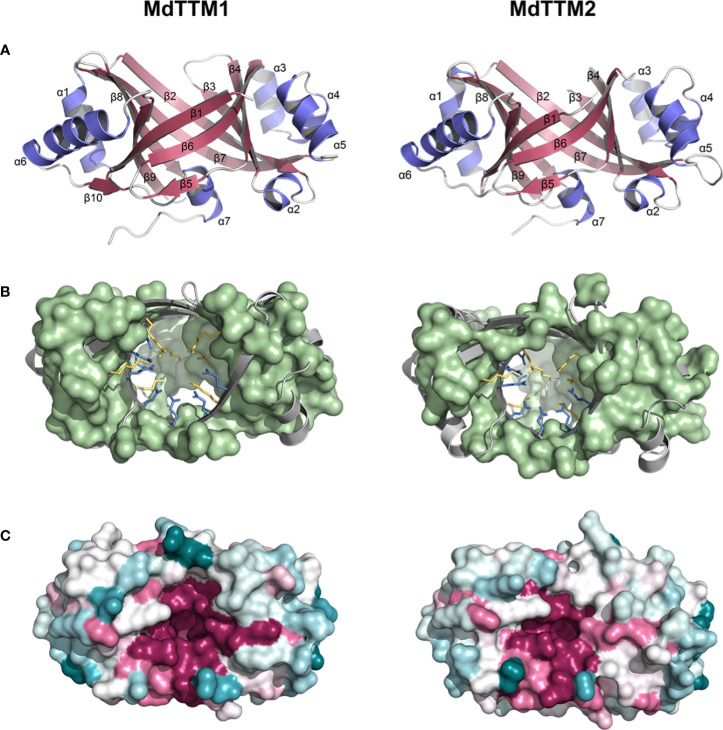
Protein prediction analysis of MdTTM1 and MdTTM2. **(A)** Overall fold view of MdTTM1 and MdTTM2. **(B)** Basic and acidic residues of the inside tunnel of MdTTM1 and MdTTM2. Basic and acid residues were presented with yellow and cyan sticks, respectively. Hydrophobic residues were presented with a light green surface. **(C)** Surface models of MdTTM1 and MdTTM2. Different colors represent the conservation level of the proteins. MdTTM1 and MdTTM2 were modeled with AlphaFold 2.0. Conservation analyses were performed on Consurf Server (https://consurf.tau.ac.il/consurf_index.php), and images were created with PyMOL (ver. 2.5).

In order to get an insight into the cyclizing ATP mechanism of MdTTM1 and MdTTM2, computational methods like the prediction of protein structure and docking simulation of ATP to enzyme were applied. AlphaFold 2.0 was used to predict the enzyme structures of MdTTM1 and MdTTM2 (**Figure 4A**). The structures of these two proteins are similar to each other in general. They all exhibited typical TTM features and β-sheets that formed a catalytic tunnel, but with delicate differences. The topologically closed tunnels in MdTTM1 and MdTTM2 were both enclosed by nine anti-parallel β-sheets, except for two successive β-sheets, β4 and β5, which shared the same orientation like having been cut off from one long strand. The α-helixes from MdTTM1 and MdTTM2 enzymes showed a high consistency—all armed with seven α-helixes and wherein α2, 3, 4, 5, and α1, 6 were located on two sides of the tunnel and α7 was laid on the opening of a barrel nearby the C-terminal. There were some delicate structural differences as well. The β1 in MdTTM1 was composed of more residues, and MdTTM1 harbored one extra β-sheet between α6 and α7.

The residue distribution inside the barrel also provided astonishing uniformity even between species according to the alignment of tertiary structures. The hydrophobic parts of MdTTM1 and MdTTM2 were mostly spread around the barrel, and several hydrophobic residues from different proteins were situated in nearly overlapping positions. Acidic residues inside the barrel were laid on regions around the β1 fold for MdTTM1 and MdTTM2 (**Figure 4B**). Especially the five conserved residues, E10, E12, E89, E91, and E165 in MdTTM1, emerged at similar positions of MdTTM2, which resulted in approximate regions to coordinate Mn^2+^. The analogous situation was also suitable for positive residues—seven positively charged residues laid on parallel positions of both MdTTM1 and MdTTM2 (**Figure 4B**). The Consurf Server provided tertiary structures colored according to residue conservation after alignment with sequences from the UniRef 90 database. It was evident that the catalytic centers were constructed with the most conserved residues (**Figure 4C**). Even the side chains of limited residues with less conservation on the tunnel were pointing against the tunnel axis (**Supplementary Figures 1A, B**).

**Figure 5:**
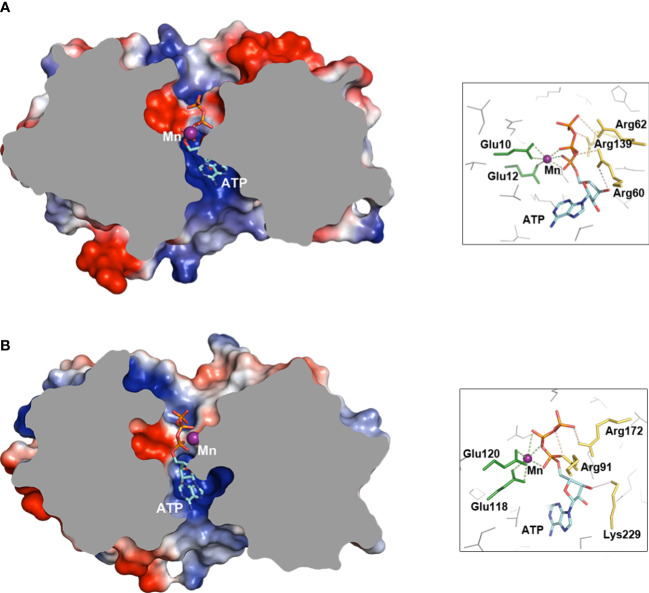
Docking simulations of ATP to MdTTM1 and MdTTM2. **(A)** Docking pose of substrate ATP to MdTTM1, free energy -8.3 (kcal/mol). **(B)** Docking pose of substrate to MdTTM2, free energy -8.4 (kcal/mol). The proteins are truncated from the middle to show the poses of ATP, and the insets show candidate functional residues that bind Mn and ATP. The icons on the right of proteins display functional residue coordinating ligands. The green broken lines represent coordination bonds to Mn^2+^ (distance range, 2.2 to 2.8 Å). The light orange broken lines show the H-bonds between a protein and a ligand shorter than 3.5 Å. The light pink broken lines indicate a nucleophilic attack vector. ATP docking simulations were performed using AutoDock Vina (ver. 1.2.0), and images were created with PyMOL (ver. 2.5).

Within all the above-mentioned similarities, the functional residues serving to cyclization presented at conserved positions on the simulated docking poses of ATP to enzymes that were deduced by Autodock Vina. Overall, the adenine moieties were both at the side nearby the α7 and triphosphate parts on the other side of the barrels surrounding Mn^2+^ (**Figure 5**). As to functional residues, both MdTTM1 and MdTTM2 provided two conserved adjacent glutamic acid to coordinate Mn^2+^, and there were residues Arg60, Arg62, and Arg139 in MdTTM1 H-bonding phosphate oxygen, and the basic residues were Arg91 and Arg172 in MdTTM2. Moreover, both MdTTM1 and MdTTM2 produced one arginine nearby the ribose O3′ of ATP as a candidate base, which made it possible to deprotonate O3′ and sequentially trigger cyclization. Although the docking gestures of ATP and the functional residues in the two enzymes were similar. differences also existed—for example, Mn^2+^ in the MdTTM1 barrel seemed to coordinate phosphate oxygen of P^α^ to make it more electropositive, which benefits a nucleophilic attack (**Figure 5B**), while the same target was accomplished by both Mn^2+^ and positively charged residues in MdTTM2 (**Figure 5A**).

### The phosphohydrolase activity of MdTTM1 and MdTTM2

CYTH is an ancient domain named by combining adenylyl cyclase and thiamine triphosphatase (ThTPase) (Iyer and Aravind, 2002). It was proposed that CYTH could be considered as a branch of TTM (Gong et al., 2006). However, the ThTPase and RNA triphosphatase activities were mainly found in mammals and yeast (*S. cerevisiae*). We have demonstrated that MdTTM1 and MdTTM2 have AC activity, but TTM was their annotations in the NCBI database, so to check whether they could present phosphohydrolase activity like proteins annotated as TTM in *A. thaliana* and *B. distachyon*, the hydrolysis activity of MdTTM1 and MdTTM2 on organic or inorganic phosphohydrolase was tested.

**Figure 6:**
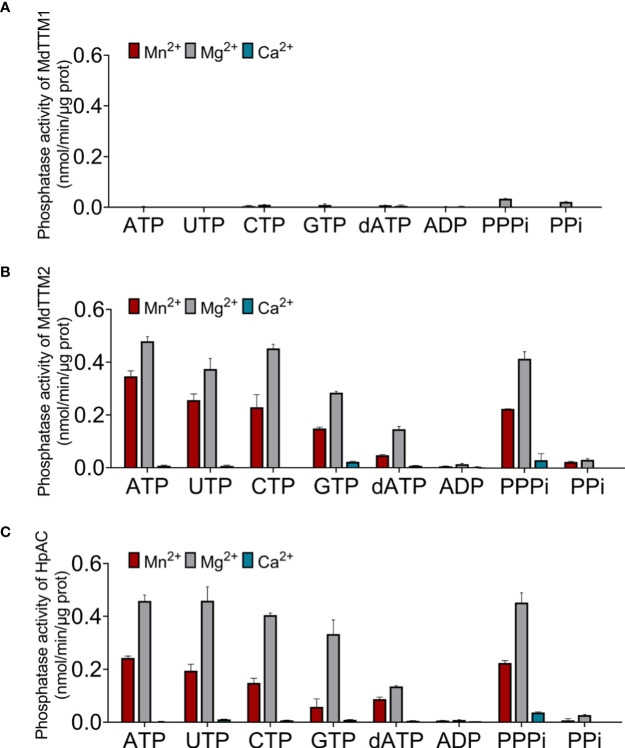
Phosphate hydrolysis activity of MdTTM1 **(A)**, MdTTM2 **(B)**, and HpAC1 **(C)** concomitant within Mn^2+^, Mg^2+^, and Ca^2+^. The Pi generated from a reaction mixture, harboring 50 μg of purified protein, 2.5 mM divalent cation, and with ATP as the substrate, was determined with Malachite Green Phosphate Assay Kits (POMG-25H). At least three independent replications were performed. Error bars represent standard deviation.

The catalytic capacity was determined by monitoring the Pi concentration in the catalytic system. As shown in **Figure 6**, MdTTM1 and MdTTM2 tended to be categorized into different types. MdTTM2 showed a powerful hydrolysis capacity for both organic and inorganic triphosphate substrates involving ATP, UTP, CTP, GTP, and PPPi (**Figure 6B**). Mg^2+^ rather than Mn^2+^, which is most helpful for AC activity, could assist MdTTM2 to approach its highest ATP hydrolysis activity of 0.48 nmol/min/μg, which was around 5.8 × 10^4^ times higher than its cyclase activity. Mn^2+^, as the co-factor, could also provide effective support for hydrolyzing ATP. Its catalytic activity reached 0.35 nmol/min/μg. Ca^2+^, on the other hand, eliminated the hydrolysis activity of these enzymes. Meanwhile, MdTTM1 unfolded an exceedingly distinct peculiarity in such a way that it nearly did not possess phosphohydrolase activity for either organic or inorganic phosphate substrate in the presence of Mn^2+^ or Ca^2+^. Mg^2+^ was capable of improving the hydrolysis activity, especially, to inorganic triphosphate and pyrophosphate. The catalytic activity of hydrolyzing triphosphate reached 0.034 nmol/min/μg, which was still extremely weaker than the PPPase activity of MdTTM2. The improvement of hydrolysis activity to organic triphosphate and pyrophosphate was restricted. The ATPase activity reached 0.0017 nmol/min/μg, which was still 95 times than the AC activity (**Figure 6A**). Interestingly, this grouping mode corresponded with the cyclase activity classification mentioned before, indicating that plant TTMs possibly consisted of two types.

Given the high conservation of HpAC1 and MdTTMs, it was thought that HpAC1 could hydrolyze phosphate. The result of the catalytic test approved this speculation. HpAC1 could hydrolyze both organic and inorganic triphosphates like MdTTM2. Its ATPase activity achieved 0.46 nmol/min/μg, which was much higher than its AC activity. Besides this, HpAC1 also preferred Mg^2+^ as the co-factor and was disabled with Ca^2+^ (**Figure 6C**) for hydrolyzing triphosphates. Thus, although HpAC1 was classified into class IV AC, it probably tended to be the triphosphate hydrolase moonlighting the AC activity as well as MdTTM2.

## Discussion

Since Earl Wilbur Sutherland discovered cAMP while researching how adrenaline affected the breakdown of glycogen into glucose in hepatocytes (Sutherland and Rall, 1957), substantial studies on AC in mammals and microorganisms have been performed, including sequences, classification, biological function, and crystal structure analysis, which promoted the unmasking catalytic mechanism development of AC (Tesmer et al., 1997; Guo et al., 2005; Steegborn et al., 2005). However, hindered by the extremely limited levels of cAMP in plant tissues (Spiteri et al., 1989), progress on plant ACs could not proceed smoothly as it was in animals and microorganisms. It was not until 2001 that the first plant AC, ZmPSiP, was experimentally confirmed in maize (Moutinho et al., 2001), accelerating the plant AC studies. In the next 20 years, particularly the recent 10 years, another 14 plant ACs have been verified from herbaceous plants, comprising of eight ACs from *A. thaliana* (Ruzvidzo et al., 2013; Al-Younis et al., 2015; Al-Younis et al., 2018; Chatukuta et al., 2018; Bianchet et al., 2019; Dias et al., 2019; Ruzvidzo et al., 2019; Al-Younis et al., 2021; Sehlabane et al., 2022), one from *M. polymorpha* (Kasahara et al., 2016), one from *N. tabacum* (Ito et al., 2014), two from *B. distachyon* (Świeżawska et al., 2020; Duszyn et al., 2022), one from *Z. mays* (Yang et al., 2020), and one from *Hippeastrum* × *hybridum* (Świeżawska et al., 2014). Meanwhile, we identified MdTTM1 and MdTTM2, which were the first enzymes to demonstrate AC activity as discovered in woody plants, thus providing more basis for the wide distribution of ACs in plants.

MdTTM1 and MdTTM2 displayed some parallel catalytic properties with other plants’ ACs. The most obvious common characteristic was their restricted catalytic activities. From the aspect of catalytic efficiency, although they exhibited cyclase ability, their dominant catalytic capability was mostly on cutting off the loose terminal phosphate, which was the hydrolyzing activity. A similar situation likewise appeared in the hydrolytic activities of plant ACs, such as BdTTM3 and the HpAC1 categorized as class IV ACs though. Whereas the class IV ACs from microorganisms *A. hydrophila* and *Y. pestis* display much more powerful AC activities which could reach up to 1,313 and 6,300 nmol/min/mg, respectively (Sismeiro et al., 1998; Smith et al., 2006). In addition, ACs in other classes also perform a forceful catalyzing activity, like the class -I AC in *E. coli* with an *in vitro* catalytic efficiency of 700 nmol/min/mg (Yang and Epstein, 1983) and 1,000 nmol/min/mg for class III AC from the yeast *S. cerevisiae* (Shima et al., 1997). Thus, HpAC1 is more like a hydrolase than a cyclase, and the AC activity of MdTTM1, MdTTM2, HpAC1, and BdTTM3 seemed to be an additional property. In fact, all the reported plant ACs so far exhibit limited AC activities. The *in vitro* AC activity of plant ACs was universally lower than 1 nmol/min/mg. Among them, AtCIAP, AtNCED3, NbAC, AtPPR, AtKUP5, AtKUP7, ZmPSiP, ZmRPP13-LK3, MpCAPE-AC, and BdTTM3 showed a similar AC activity level with MdTTMs, ranging from 0.001 to 0.02 nmol/min/mg. The AC activities of BdGUCD1 and HpAC1 were higher, about 0.077 and 0.3 nmol/min/mg.

It is not surprising that the plant ACs seem not to be the specific ACs but instead likely possess functions of other enzymes with corresponding structures. The reported plant ACs tend to be of the “moonlighting” protein category, displaying at least two functions and various protein structures. Typically, ZmPSiP, as the first confirmed plant AC, is disguised in the disease resistance protein. AtKUP5 was a potassium transporter that could rescue K^+^ transport-deficient *S. cerevisiae* mutant. AtKUP7 functions vitally on *A. thaliana* potassium ion absorption and translocation, particularly one that is involved in K^+^ transferring from roots to shoots under K^+^-limited conditions. Unlike these proteins, MdTTM1 and MdTTM2 are the proteins that involve a phosphate mechanism from the beginning. Inorganic phosphate is the original substrate for TTM, and as of now, hydrolyzing organic and inorganic phosphate is still the main activity for most TTMs, including MdTTM1 and MdTTM2. Even the ThTPase activity from animal tissues and Cet1 RNA triphosphatase activity from *S. cerevisiae* are still hydrolyzing phosphate, but inside the tunnels of MdTTM1 and MdTTM2, the functionally charged residues are capable of coordinating the ATP–Mn complex, and nearby the O3′ of ATP locates one positively charged residue which could accomplish deprotonation and then initiate the cyclization process, or the initiation process could also be achieved by introducing another divalent cation. Consequently, MdTTM1 and MdTTM2 displayed AC activity. It could not be neglected that the AC activity is weak, which might be because the gesture of ATP–Mn coordinating with protein was not the most appropriate way for cyclization; the ATP–Mn complex was hard to get to the imaginary position or the impact from a powerful hydrolyzing activity. Given the widespread TTM homologs, it is not surprising to speculate that they played vital roles in plant mechanism. Świeżawska et al. found that the expression levels of HpAC1 and cAMP content were both upregulated under certain stress conditions, but it still lacked the evidence to demonstrate HpAC1 synthesizing cAMP *in vitro* in response to stress conditions. BdTTM3 was speculated to participate in the mechanism underlying responses to wounding stress in *B. distachyon* leaves as well, and AtTTM3 was involved in the root development of *A. thaliana*. In view of the parallel features on structure and catalysis between MdTTMs and these proteins, MdTTMs might play analogous roles in stress resistance and the growth of apple. Of course, the relationship between MdTTMs and cAMP in plant tissues remain questionable. Perhaps MdTTMs could upregulate a microlocal cAMP content change and then involve various metabolism pathways. Overall, the “part-job” character of plant ACs is dominant, which implies that plant proteins involved in phosphate metabolism are available for AC activity determination—for instance, given that a diacylglycerol kinases (DGK) activity from cultured *Catharanthus roseus* cells could accept both ATP and GTP as substrates (Wissing and Wagner, 1992), the AC activity of AtDGK4 was also examined after the confirmation of the GC activity of AtDGK4 (Dias et al., 2019).

TTM proteins are found widely in bacterial, archaeal, and eukaryal taxa with multiple substrate specificities and enzymatic activities. Although plenty of TTMs is annotated as AC, particularly in archaebacteria, most of them are not supported by experimental data (Keppetipola et al., 2007). Iyer and Aravind (2002) proposed inorganic tripolyphosphate as the initial substrate for TTMs, while other enzyme activities involving AC, mRNA triphosphatase, and ThTPase are secondary acquisitions. The ThTPase activity belongs to partial metazoan TTMs, while TTMs with mRNA triphosphatase were found in fungi and protozoans (Song et al., 2008). Primitive hydrolase activity appears to have a major role for plant TTMs, while AC activity was acquired through several functional residues. There are three genes in *A. thaliana* genome annotated as TTMs, which present hydrolase activity. AtTTM3 possesses tripolyphosphatase activity and a weaker nucleotide triphosphatase (Ung et al., 2017), while AtTTM1 and AtTTM2 display a higher preference for pyrophosphate (Moeder et al., 2013; Ung et al., 2014). All three AtTTMs exhibit a weaker affinity for nucleotide triphosphates, but no AC activity has been detected. On the contrary, MdTTM2 and HpAC1 could hydrolyze both organic and inorganic triphosphates, simultaneously cyclizing ATP and hydrolyzing pyrophosphate with pretty low efficiency. MdTTM1, on the other hand, possesses similar types of enzymatic activities as MdTTM2, but all with very limited activity. So, it seems that MdTTMs possess two enzymatic catalysis types. Nevertheless, MdTTM1, MdTTM2, and HpAC1 are closer to AtTTM3 than AtTTM1 and AtTTM2. They all harbor a conserved EXEXK motif which, however, is TYILK in AtTTM1 and AtTTM2 and does not provide an additional N-terminal uridine kinase domain. In fact, there is a different voice on the HpAC1 classification. The results of the activity assays conducted by Kleinboelting et al. (2020) suggested a dominant hydrolysis activity for triphosphate substrates, especially PPPase activity. Given the typical TTM crystal structure and PPPase activity of HpAC1, they proposed to rename it to HpPP1 (polyphosphatase 1) or HpTTM1.

It was generally accepted that plant tissues possess a trace concentration of cAMP, which was even debatable in terms of whether it was enough for signal transduction or not. Spiteri et al. (1989) found cAMP to be existing in *Lactuca sativa*, *Helianthus annuus*, *Oryza sativa*, *Pinus pinaster*, and *N. tabacum* with content levels of under 5 pmol/g. Water or GA3 immersion could upgrade the cAMP level in barley seeds from 0.2 to 2 nmol/g (Miller and Galsky, 1974). However, reports about cAMP contents in apple and pear are extremely limited, except that our lab has previously determined the cAMP content in apple fruit, which reached 336 pmol/g FW (Liu and Wang, 1991). It is surprising that, among the 14 horticultural plants where cAMP has been detected, Chinese jujube exhibited a mighty accumulation capacity of cAMP in mature fruit, which agreed with the results of Cyong and Hanabusa (1980), indicating that there probably is a special mechanism in jujube fruit. However, for most plants, deficiency of high-performance AC could be one important reason for the strictly finite cAMP content, especially in view of the somewhat limited AC activity and specificity of all plant ACs verified by now. Nevertheless, the AC activity of proteins from a woody plant further demonstrates the wide distribution of plant ACs, which might contribute to research on the evolutionary relationship and signal transduction of plant ACs and perhaps assist in upgrading the cAMP content in fruit, thus subsequently diversifying the nutritional ingredients.

## Materials and methods

### Alignment and analysis of candidate AC proteins

The amino acid sequences of candidate AC proteins in apple (MdTTMs) were screened out by aligning with the sequence of HpAC1 (ADM83595.1) with the help of protein–protein BLAST (Basic Local Alignment Search Tool) in NCBI (https://www.ncbi.nlm.nih.gov/) (Johnson et al., 2008). Based on acquired information of sequences, the software DNAMAN was used for multiple sequence alignment, and MEME suite was used to identify conserved motifs by setting eight possible candidate motifs to target (Bailey et al., 2015).

### Protein purification

To obtain the purified MdTTMs and HpAC1, their nucleotide coding sequences were synthesized by General Biosystems (Anhui) Co., Ltd., and inserted into the pET-28a vector. The recombinant vectors were transformed into BL21 (DE3)-competent Escherichia *coli*. Positive colonies were selected by a plate coating method and inoculated into Luria Broth (LB) medium for protein over-expression at 20°C for about 10 h after induction by 1 mM isopropyl-1-thio-β-D-galactopyranoside (IPTG). Ni-affinity chromatography was used to purify rough extracted protein after sonication according to the manufacturer’s protocol. In addition, SDS–polyacrylamide gel electrophoresis was applied to reveal protein purity.

### *In vitro* enzymatic properties of MdTTMs

The enzymatic property of MdTTMs and HpAC1 was determined by testing the cAMP content and Pi as reaction products. The 500-μl reaction system comprising of 50 mM TRIS-HCl (pH 7.5), 0.5 mM ATP, 2.5 mM divalent cation, and 50 μg purified protein was prepared and incubated at 30°C. The reactions were stopped by protein denaturation at 95°C. The cAMP content in the product solution was determined by Plant cAMP ELISA Kit (LE-Y1602, Hefei Lai Er Bio-Tech Co., Ltd). The released free phosphate in solution was measured by the Malachite Green Phosphate Assay Kits (POMG-25H).

### Complementation of *cyaA* mutation in *E. coli* SP850 strain

E. coli *cyaA*-mutant SP850 strain [lam-, el4-, relA1, spoT1, cyaA1400 (:kan), thi-1] (Shah and Peterkofsky, 1991), deficient in AC, was used to determine the AC activity *in vivo*. Recombinant prokaryotic expression vectors were transformed into SP850 strain by heat shock at 42°C for 90 s. Bacteria grown in LB media containing 50 µg/ml kanamycin and 100 µg/ml ampicillin were induced with 0.5 mM IPTG, Beijing Solarbio Technology Co., Ltd.) when they reached an OD_600_ of 0.6. After 4 h of induction, the bacteria were streaked on MacConkey agar to observe color changes.

### Structural analysis and molecular docking simulation

The model of MdTTM2 was obtained from AlphaFold Protein Structure Database (https://alphafold.ebi.ac.uk/), and the model of full-length MdTTM1 was predicted with AlphaFold 2.0 (Jumper et al., 2021). Conservation analyses of MdTTM1 and MdTTM2 were conducted by automatically aligning the primary structure with UniRef90 database (Suzek et al., 2015) on the Consurf Server (Ashkenazy et al., 2016). The ligand model was downloaded from Protein Data Bank (http://www.wwpdb.org/). Molecular docking of ATP to predicted proteins was accomplished by Autodock Vina (ver. 1.2.0) (Trott and Olson, 2009). PyMOL (ver. 2.5) (DeLano, 2002) was used to analyze the ATP docking pose and create docking images.

## Data availability statement

Publicly available datasets were analyzed in this study. These data can be found here: https://www.ncbi.nlm.nih.gov/search/all/?term=LOC103400052 https://www.ncbi.nlm.nih.gov/search/all/?term=LOC103428055 https://www.ncbi.nlm.nih.gov/search/all/?term=LOC103426573.

## Author contributions

Conceptualization: ML, ZL, and YY; methodology: YY, SC, YN, PL and LLW; writing—original draft preparation: YY; writing—review and editing: ZL, ML, and LXW; visualization: YY, ZL, and LXW; supervision: ZL, ML, PL and LLW; and funding acquisition: ML. All authors contributed to the article and approved the submitted version.

## Funding

This work was funded by the National Key R&D Program of China (2019YFD1001605, 2018YFD1000607, and 2020YFD1000705), the Natural Science Foundation of Hebei Province (C2020204082), Subsidy Funds for Hebei Jujube Industry Technology Research Institute after Operation Performance (205676155H), Hebei Province Funding Project for Overseas Talents (C20210114), the Youth Fund of Hebei Province Natural Science Foundation (C2021204101), and the National Natural Science Foundation of China (32101542).

## Acknowledgments

The authors wish to thank everyone in the lab for their excellent technical assistance.

## Conflict of interest

The authors declare that the research was conducted in the absence of any commercial or financial relationships that could be construed as a potential conflict of interest.

## Publisher’s note

All claims expressed in this article are solely those of the authors and do not necessarily represent those of their affiliated organizations, or those of the publisher, the editors and the reviewers. Any product that may be evaluated in this article, or claim that may be made by its manufacturer, is not guaranteed or endorsed by the publisher.
