# Supplementary material for: Two triphosphate tunnel metalloenzymes from apple exhibit adenylyl cyclase activity
Source: Front Plant Sci. 2022 Oct 6;13:992488. doi: 10.3389/fpls.2022.992488 (PMC9582125; doi:10.3389/fpls.2022.992488)
Supplement: Supplementary file 1 [file Presentation_1.pptx]

## Slide 1
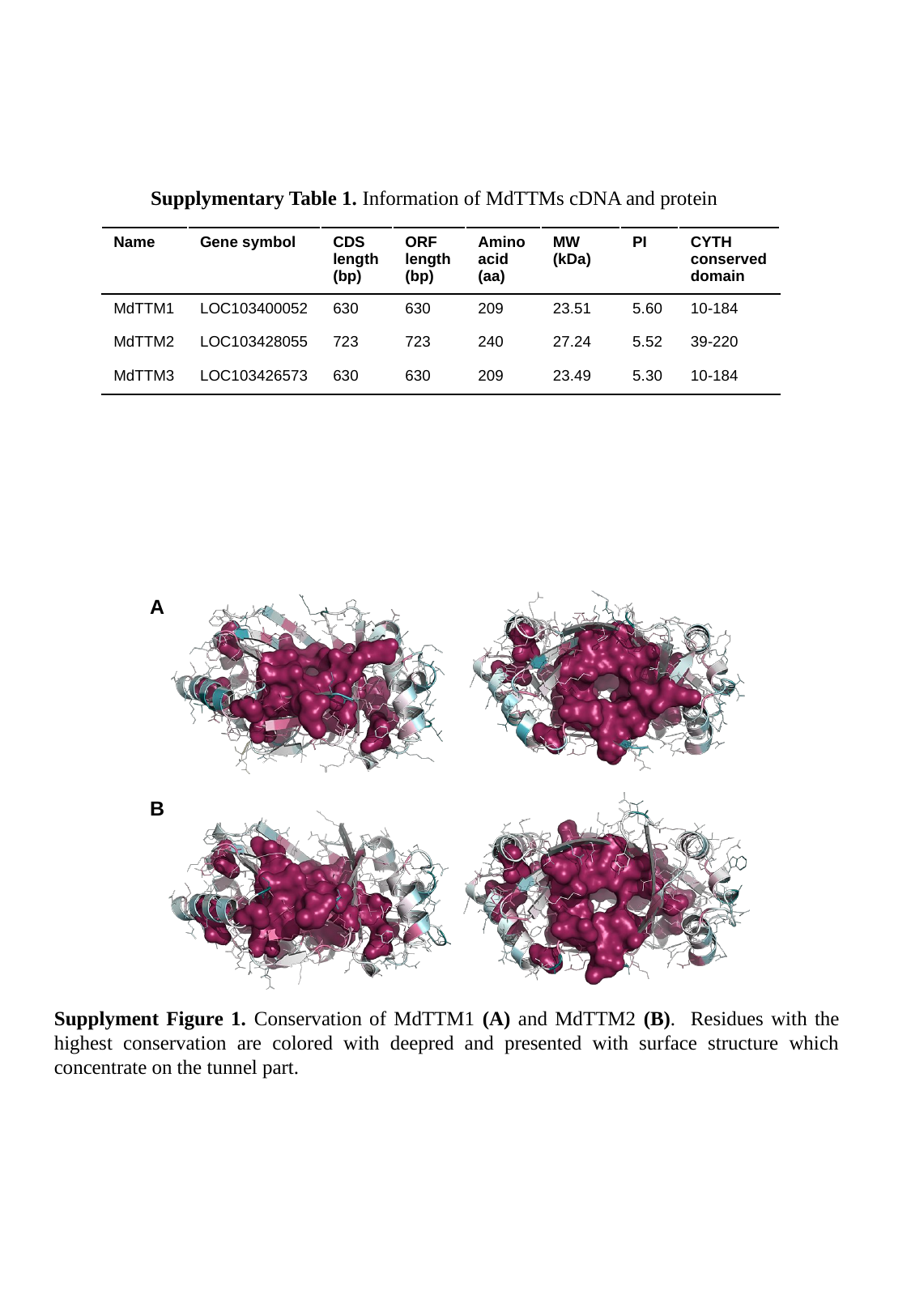

Supplymentary Table 1. Information of MdTTMs cDNA and protein
| Name | Gene symbol | CDS length (bp) | ORF length (bp) | Amino acid (aa) | MW (kDa) | PI | CYTH conserved domain |
| --- | --- | --- | --- | --- | --- | --- | --- |
| MdTTM1 | LOC103400052 | 630 | 630 | 209 | 23.51 | 5.60 | 10-184 |
| MdTTM2 | LOC103428055 | 723 | 723 | 240 | 27.24 | 5.52 | 39-220 |
| MdTTM3 | LOC103426573 | 630 | 630 | 209 | 23.49 | 5.30 | 10-184 |
A
B
Supplyment Figure 1. Conservation of MdTTM1 (A) and MdTTM2 (B). Residues with the highest conservation are colored with deepred and presented with surface structure which concentrate on the tunnel part.

## Slide 2
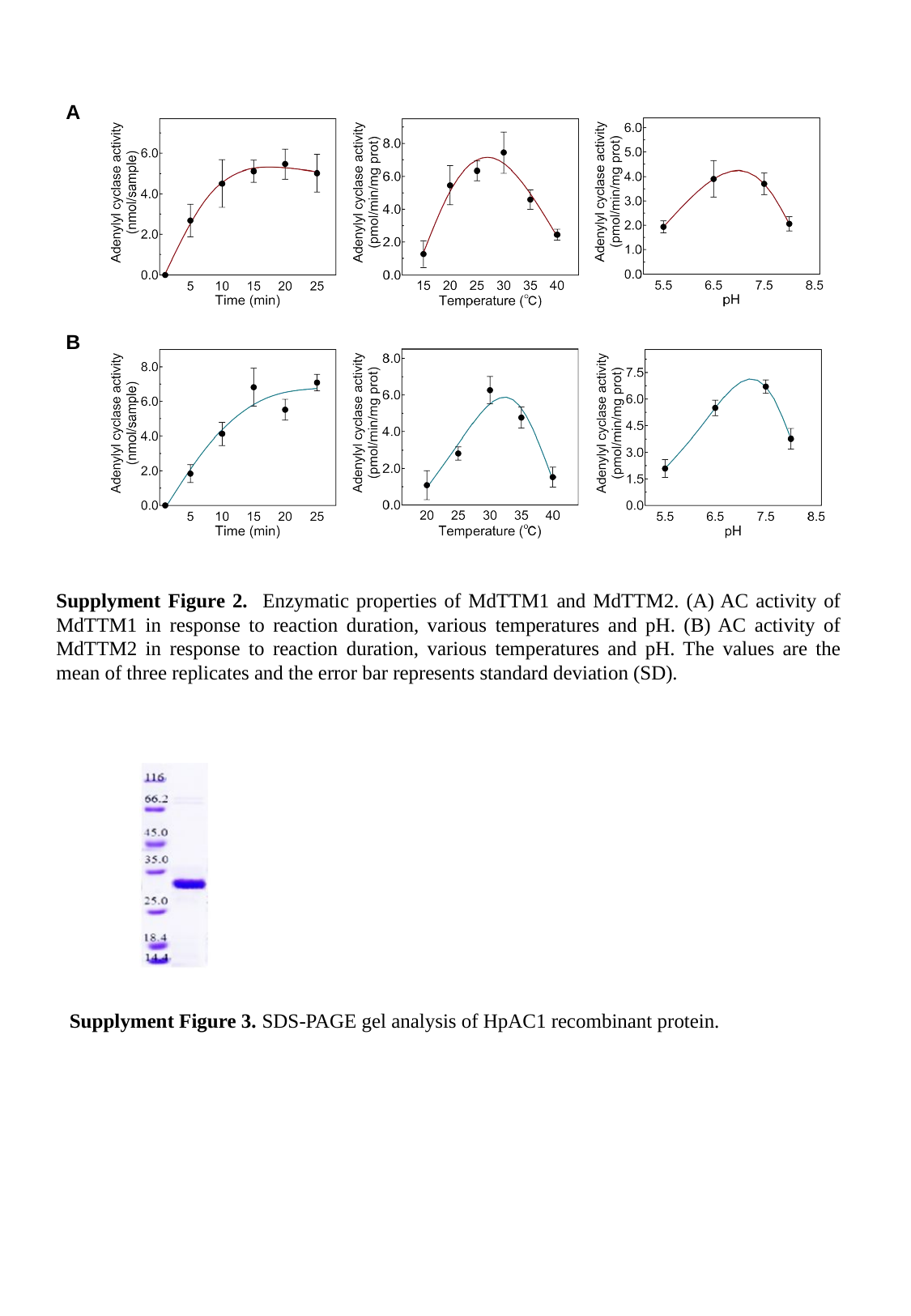

A
B
Supplyment Figure 2. Enzymatic properties of MdTTM1 and MdTTM2. (A) AC activity of MdTTM1 in response to reaction duration, various temperatures and pH. (B) AC activity of MdTTM2 in response to reaction duration, various temperatures and pH. The values are the mean of three replicates and the error bar represents standard deviation (SD).
Supplyment Figure 3. SDS-PAGE gel analysis of HpAC1 recombinant protein.

## Slide 3
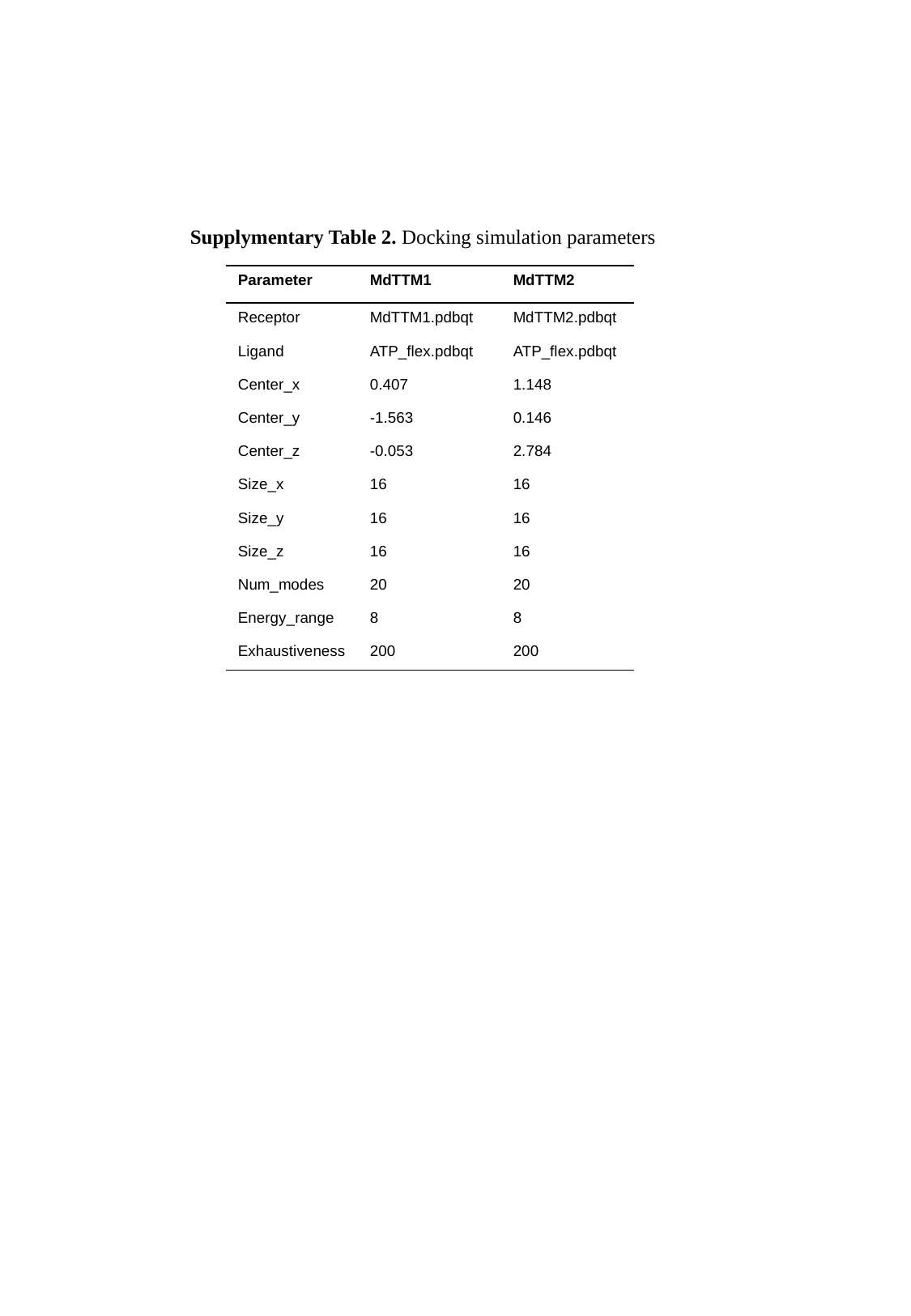

Supplymentary Table 2. Docking simulation parameters
| Parameter | MdTTM1 | MdTTM2 |
| --- | --- | --- |
| Receptor | MdTTM1.pdbqt | MdTTM2.pdbqt |
| Ligand | ATP\_flex.pdbqt | ATP\_flex.pdbqt |
| Center\_x | 0.407 | 1.148 |
| Center\_y | -1.563 | 0.146 |
| Center\_z | -0.053 | 2.784 |
| Size\_x | 16 | 16 |
| Size\_y | 16 | 16 |
| Size\_z | 16 | 16 |
| Num\_modes | 20 | 20 |
| Energy\_range | 8 | 8 |
| Exhaustiveness | 200 | 200 |
